# Recent Advances in Porcine Reproductive and Respiratory Syndrome Virus NADC30-Like Research in China: Molecular Characterization, Pathogenicity, and Control

**DOI:** 10.3389/fmicb.2021.791313

**Published:** 2022-01-11

**Authors:** Ying Yu, Qiaoya Zhang, Zhi Cao, Yan-Dong Tang, Dasong Xia, Gang Wang, Hu Shan

**Affiliations:** ^1^Department of Preventive Veterinary Medicine, College of Veterinary Medicine, Qingdao Agricultural University, Qingdao, China; ^2^State Key Laboratory of Veterinary Biotechnology, Chinese Academy of Agricultural Sciences, Harbin Veterinary Research Institute, Harbin, China; ^3^College of Veterinary Medicine, Shandong Agricultural University, Taian, China

**Keywords:** PRRSV, NADC30-like, genetic diversity, pathogenicity, control

## Abstract

The name porcine reproductive and respiratory syndrome virus (PRRSV) NADC30-like was first coined in 2015. It originated from the NADC30 strain that was introduced into China by importing breeding pigs and has since undergone mutations or recombination, resulting in variant viruses. Following widespread outbreaks in China in recent years, these NADC30-like strains have presented major health challenges in swine production systems. Outcomes induced by PRRSV NADC30-like infection are highly variable, ranging from inapparent to severe, depending on the recombination between NADC30 and field PRRSV strains prevalent in swine farms. Vaccines and strict biosecurity measures have been explored to fight this disease; however, current PRRSV commercially modified-live virus vaccines (MLVs) have the potential to revert to virulence and only provide limited or no cross-protection efficacy against NADC30-like strains. PRRSVs will remain an ongoing challenge to the swine industry until safe and effective vaccines or antiviral reagents are developed.

## Introduction

Porcine reproductive and respiratory syndrome (PRRS) is a highly contagious disease caused by PRRS viruses (PRRSVs), which are small, enveloped, positive single-stranded RNA viruses in the genus *Porartevirus*, family *Arteriviridae*, and order *Nidovirales* (Adams et al., 2017). Currently, PRRSV exists as two distinct virus species, i.e., PRRSV-1 (known as the European genotype, type strain Lelystad) and PRRSV-2 (known as North American genotype, strain VR-2332), with both species sharing ∼60% nucleotide identity at the genomic level and are subjected to frequent mutation and viral recombination events (Nelsen et al., 1999; Adams et al., 2017).

Porcine reproductive and respiratory syndrome virus contains an infectious RNA genome in a proteinaceous nucleocapsid, surrounded by a lipid layer including five or six enveloped structural proteins. The genome of PRRSV contains ∼15 kb nucleotides, with cap structure at the 5′ end and a poly (A) tail at the 3′ end, and contains approximately 11 open reading frames (ORFs) (1a, 1b, 2a, 2b, 3, 4, 5a, 5, 6, 7, and a short transframe ORF) expressed from genomic and subgenomic (sg) mRNAs (sgmRNAs) (Conzelmann et al., 1993). ORF1a and ORF1b are translated as large polypeptides, pp1a and pp1ab, which are then proteolytically processed into the nonstructural proteins (nsp) of PRRSV (nsp1α, nsp1β, nsp2, nsp2TF, and nsp3–nsp12); ORFs 2–7 encode the structural proteins of PRRSV (GP2a, E, GP3–GP5, M, and N) (Meulenberg et al., 1997; Snijder and Meulenberg, 1998; Snijder et al., 2013).

Since the highly pathogenic PRRSV strains were seeded in swine farms in China in 2006 (Li et al., 2007; Tian et al., 2007; Tong et al., 2007), the clinical prevalence of PRRSV has become complicated. To make matters worse, the PRRSV NADC30 strain began to infect Chinese pigs in 2014 (Zhao et al., 2015; Zhou et al., 2015; Li C. et al., 2016; Li Y. et al., 2016). Currently, the recombination characteristics of NADC30 contribute to the emergence of variant NADC30-like viruses, as well as the highly variable clinical symptoms, ranging from inapparent to severe symptoms (Zhao et al., 2015; Zhou et al., 2015; Wang et al., 2018). Herein, we review the current information related to the novel PRRSV strains (NADC30-like) in China in terms of their molecular characterization (genetic diversity) and pathogenicity (virulence), and the questionable efficacy of current vaccines against this disease.

## Molecular Characterization (Genetic Diversity of Viruses)

**FIGURE 1:**
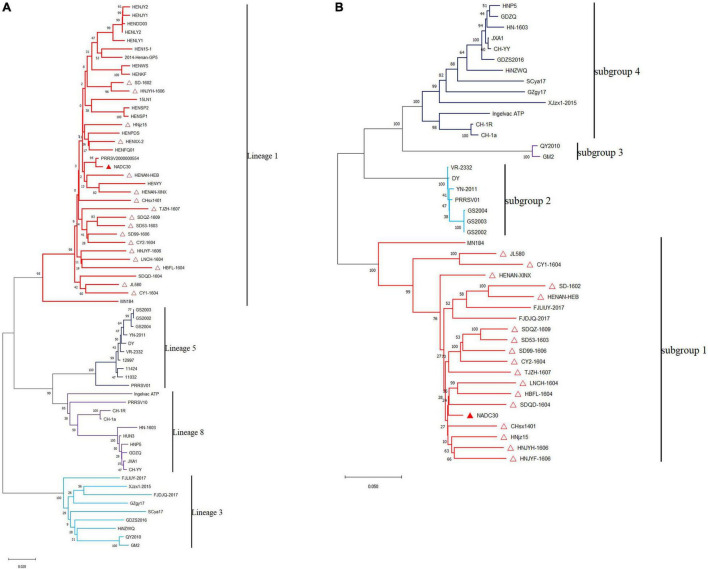
Phylogenetic tree based on PRRSV ORF5 **(A)** and nsp2 gene **(B)** sequences. The phylogenetic tree was constructed using the neighbor-joining method in MEGAX64 with 1,000 bootstrap replicates. Multiple sequence alignments were generated using MUSCLE. NADC30 (GenBank number: JN654459) and 17 references NADC30-like PRRSV strains are indicated by closed red triangles and open red triangles, respectively.

The molecular markers of NADC30 are 131-aa discontinuous deletions in nsp2, including a 111-aa deletion at position 322–432, a 1-aa deletion at position 483, and a 19-aa deletion at position 504–522 corresponding to the NADC30 complete sequence (Brockmeier et al., 2012). All the isolates, called NADC30-like strains, also have the same molecular markers and belong to lineage 1 based on GP5 analysis (Figure 1A) and subgroup 1 based on nsp2 analysis (Figure 1B) (Shi et al., 2010).

Recombination breakpoints occurring in genes encoding nsps and/or minor structural proteins contribute to the variant pathogenicity of individual isolates, including highly pathogenic, moderate virulent, or mild strains, depending on their other recombination positions with variant local PRRSV strains (Zhao et al., 2015; Zhou et al., 2015; Sun et al., 2016; Wang et al., 2018; Zhang et al., 2019). Currently, there is no reasonable explanation for the random recombination of variant NADC30-like isolates; the postulated reason is the pressure of the complicated PRRSV pool in a swine farm pushes the invading NADC30 strain to survive *via* a new strategy. Those recombination breakpoints can be found in the 5′ UTR, nsp1-9, nsp12, ORF3, and ORF5-3′ UTR (Guo et al., 2019; Zhang et al., 2019; Zhao et al., 2020).

### Outcomes Produced by Porcine Reproductive and Respiratory Syndrome Virus NADC30-Like Infection Are Highly Variable

All the NADC30-like isolates reported from swine farm outbreaks of PRRS are characterized by abortions and stillbirth in pregnant sows, as well as high fever, anorexia, red discoloration of the body, blue ears, and respiratory disorders in piglets. The impact of NADC30-like infection contributes to the swine farm referring to its virulence, as well as the age and immune status of the infected pigs, and the presence of concurrent infections. Pig infection experiments in the laboratory may not replicate the outcomes of clinical cases, but can be used to evaluate the pathogenicity of an isolate.

The pathogenicity of NADC30-like strains was first investigated using JL580 (accession no. KR706343), which acquired increased genetic diversity by recombining with local HP-PRRSV 09HEN1 in China at six different sites in its genome (Zhao et al., 2015; Sun et al., 2016). Six-week-old PRRSV-free piglets were inoculated intramuscularly (1 ml) and intranasally (2 ml) with JL580-F2 at 3 × 10^4.0^ TCID50 in 3 ml of Dulbecco’s modified Eagle’s medium (DMEM) per pig. These piglets were reported to develop obvious clinical symptoms from 3 days post-inoculation (DPI), e.g., higher fever, cough, anorexia, and red discoloration of the body and ears, accompanied by severe lung lesions with pulmonary consolidation, and interstitial pneumonia (Zhao et al., 2015). The outcomes induced by NADC30-like JL580 are much more severe than those of NADC30 isolates (e.g., HNjz15, CHsx1401, SC-d, SD-A19) (Brockmeier et al., 2012; Sun et al., 2016; Zhou et al., 2017; Wang et al., 2018) and CH-1a (Xue et al., 2004), and are similar to those of the HP-PRRSV strains that appeared in China from 2006 (Li et al., 2007; Tian et al., 2007; Tong et al., 2007).

Similarly, the pathogenicity of another NADC30-like isolate, HNjz15 (accession no. KT945017), with no recombination with other PRRSV strains, as compared with the pathogenicity with highly pathogenic PRRSV JAX1 strain (accession no. EF112445) (Sun et al., 2016). Six-week-old PRRSV-free pigs were inoculated intranasally with 2 ml (5 × 10^4^ TCID_50_/ml) of HNjz15 or JXA1. Piglets infected with one of the two PRRSV isolates developed typical PRRS symptoms, e.g., high fever and respiratory disorders, and the difference was that JXA1-infected piglets developed more severe clinical manifestations than the HNjz15-infected piglets (Sun et al., 2016). Analysis of the data from HNjz15- and JL580-infected piglets demonstrated that HNjz15 is less pathogenic than the JXA1 and JL580 PRRSV strains, even though those experiments were not performed at the same time.

To further clarify their pathogenic characterization, three isolates, including non-recombinant NADC30-like PRRSV (SD-A19), recombinant NADC30-like PRRSV (SC-d), and highly pathogenic PRRSV (HuN4), were used to inoculate 5-week-old SPF piglets intranasally (3 ml, 1 × 10^5^ TCID_50_/ml), respectively. The results showed HuN4 infection induced most severe PRRS disease, including rectal temperature, thymic atrophy, and interstitial pneumonia, and the SC-d isolate infection induced milder PRRS disease than that of HuN4, but more severe than that of the SD-A19 isolate (Wang et al., 2018).

Based on cumulative data, recombination is responsible for the pathogenicity variance and genetic diversity of NADC30-like PRRSVs in China, and the pathogenicity tends to be intermediate between those of the parental strains. However, we cannot find the key factors that determine viral pathogenicity.

### Lesions of Central Immune Organs

Bone marrow and the thymus are the primary lymphoid organs of the mammalian immune system, which provide suitable sites for antigen-independent B- and T-lymphocyte differentiation from stem cells. Therefore, they play important roles in humoral or cell-mediated immunity, respectively. PRRSV-induced central immune organ lesions have become a concern since severe thymic atrophy induced by highly pathogenic PRRSV infection was first reported (Wang et al., 2011). Since then, different PRRSV-1 and PRRSV-2 isolates with variable pathogenicity have been evaluated for their contribution to bone marrow and thymus lesions (Guo et al., 2013; Amarilla et al., 2016, 2017; Wang et al., 2016, 2018). PRRSV infection of susceptible CD14^+^ cells incapacitates their ability to act against microbial infection and antigen recognition, processing, and presentation to T and B cells; and PRRSV infection induced apoptosis in precursor cells, as well as CD4^+^CD8^+^ thymocytes directly by infection or indirectly *via* a bystander effect (He et al., 2012; Li et al., 2014; Wang et al., 2016). The cumulative effect leads to immunomodulation, and impairs the host’s ability to resist and/or eliminate secondary infectious agents (Wang et al., 2019, 2020).

Porcine reproductive and respiratory syndrome virus NADC30(-like) infection also induces thymic atrophy. The average ratio of thymus/body weight (g/kg) of piglets induced by recombinant NADC30-like PRRSV SC-d isolate infection was lower than that of piglets induced by non-recombinant NADC30-like PRRSV SD-A19 isolate infection, but was significantly higher than that of piglets induced by the highly pathogenic PRRSV HuN4 strain (Wang et al., 2018). The further data are sparse since the new PRRSV isolates were only reported from 2015 in China. The thymic atrophy induced by PRRSV NADC30 (-like) isolate infection suggested that their infection can cause immunomodulation by destroying the function of central immune organs. Herein, we remind investigators of the concerns regarding thymic atrophy and the mechanism by which PRRS induces these lesions because the mammalian thymus plays important roles in cell-mediated immunity.

### Current Commercial Modified Live Vaccines Against Porcine Reproductive and Respiratory Syndrome Virus NADC30-Like Isolates

Currently, different kinds of commercial PRRSV modified-live virus vaccines (MLVs) have been used widely in Chinese swine farms, and these MLVs are effective in homologous PRRSV strain challenges (Tian et al., 2009; Leng et al., 2012; Yu et al., 2015). However, NADC30-like strains inducing PRRS still cause outbreaks in vaccinated pigs, which indicates the inefficiency of current commercial PRRSV vaccines. Some reports offered evidence that current commercial PRRSV MLVs provide cross-, limited, or no protection against NADC30-like PRRSV infection, and these vaccines include VR-2332 (Boehringer-Ingelheim, Mannheim, Germany), JXA1-P80 (Pulike Biological Engineering Co. Ltd., Luoyang, China), HuN4-F112 (Harbin Weike Biotechnology Development Company, Harbin, China), GDr180 (Guangdong Yongshun Biological Pharmaceutical Co. Ltd. Guangdong, China), and TJM-F92 [Qingdao Yibang Biological Engineering Co. Ltd or Sinovet (Beijing) Biotechnology Co. Ltd. or Zoetis] as well as an attenuated low pathogenic PRRSV HB-1/3.9-P40 vaccine that was not commercialized. These commercial vaccines include classical MLVs and HP-PRRSV MLV vaccines used in swine farms in China. The inoculated PRRSV isolates include NADC30-like HNjz15 (accession number KT945017, virulent to pigs, but is less pathogenic than the JXA1 and JL580 PRRSV strains) (Sun et al., 2016), CHsx1401 (GenBank accession no. KP861625, a moderately virulent virus for piglets) (Zhou et al., 2017), HN201605 (with lower pathogenicity than HP-PRRSV strain) (Zhang et al., 2018), and v2016/ZJ/09-03 (a virulent PRRSV) (Chai et al., 2020). The cumulative results demonstrated that the pathogenicity of NADC30-like strains is complicated, and suitable PRRSV MLVs for swine farms remain to be evaluated. However, following reports of more NADC30-like strains, it is likely that PRRSV MLVs will have limited cross-protection efficacy to some NADC30-like strains (Table 1).

**TABLE 1:** The efficacy of commercial PRRSV MLVs to NADC30-like isolates.

MLVs	Company	Inoculated isolate	Protection efficacy	References
VR-2332	Boehringer-Ingelheim, Germany	HNjz15	Ineffective	Bai et al., 2016
JXA1-P80	Pulike Biological Engineering Co. Ltd	HNjz15	Ineffective	Bai et al., 2016
HuN4-F112	Harbin Weike Biotechnology Development Company	HNjz15	Ineffective	Bai et al., 2016
GDr180	Guangdong Yongshun Biological Pharmaceutical Co. Ltd	HNjz15	Ineffective	Bai et al., 2016
TJM-F92	Qingdao Yibang Biological Engineering Co. Ltd	HNjz15	Ineffective	Bai et al., 2016
Ingelvac PRRS MLV	No detail	CHsx1401	Some beneficial efficiency in shortening the period of clinical fever and in improving the growth performance	Zhou et al., 2017
JXA1-R	No detail	CHsx1401	Extremely limited cross-protection efficacy	Zhou et al., 2017
PRRSV HB-1/3.9-P40	Non-commercial	CHsx1401	Extremely limited cross-protection efficacy	Zhou et al., 2017
TJM-F92	Sinovet (Beijing) Biotechnology Co. Ltd	HN201605	Cross-protection	Zhang et al., 2018
VR-2332	Boehringer-Ingelheim	v2016/ZJ/09-03	Cross-protection in improving growth performance, decreasing the percentage of viremic pigs, and reducing gross lung lesions	Chai et al., 2020
TJM-F92	Zoetis	v2016/ZJ/09-03	Limited protection in improving growth performance, decreasing the percentage of viremic pigs, and reducing gross lung lesions	Chai et al., 2020

### Other Attempted Strategies to Control Porcine Reproductive and Respiratory Syndrome in Swine Herds

This section describes attempted strategies to control the PRRS in swine herds in China.

#### Tylvalosin Attenuated Porcine Reproductive and Respiratory Syndrome Virus-Induced Acute Lung Injury in Piglets

Tylvalosin is a member of third-generation macrolides, which has broad-spectrum activity and can exert a variety of pharmacological effects. It is often used to control the *Mycoplasma hyopneumoniae* infection in swine farms, and the premix formulation (Aivlosin 42.5 mg/g premix for medicated feedstuff) and oral powder formulation (Aivlosin 42.5 mg/g oral powder for pigs) are licensed in the EU for the treatment and metaphylaxis of EP (CVMP European Public Assessment Report [EPAR], 2013; Lopez Rodriguez et al., 2020). The Committee for Medicinal Products for Veterinary Use (CVMP) European public assessment report (EPAR) for Aivlosin is available online.^1^ In recent years, tylvalosin has been advertised because of its anti-PRRSV activities, especially for existing PRRSV NADC30-like or HP-PRRSV-like strains. In China, tylvalosin is used widely to control PRRS in many swine farms. An animal study reported that piglets received feed containing 75 ppm tylvalosin for 28 days, following challenge with PRRSV Hn isolate intranasally (2 ml, 1 × 10^4.5^ TCID_50_/ml), and then continued to receive tylvalosin medicated feed or not for another 21 days. The outcomes showed that tylvalosin attenuated PRRSV-induced clinical disease and improved growth, and similar low-intensity clinical signs were observed in these piglets compared with the piglets infected with the PRRSV Hn isolate directly (Zhao et al., 2014). Mechanistically, it was postulated that tylvalosin inhibited PRRSV-induced NF-κB activation, as well as the production of inflammatory cytokines, such as IL-6, IL-8, and TNF-α (Zhao et al., 2014).

#### Bioactive Compounds From Traditional Chinese Medicines Exhibit Anti-Porcine Reproductive and Respiratory Syndrome Virus Activities

Bioactive compounds from TCMs exhibit features of PRRSV replication inhibition; therefore, many crude TCM herbal extracts have emerged as effective alternatives in the control of PRRS, as premixes for medicated feedstuff or administration *via* intramuscular injection. The mechanisms by which bioactive compounds of TCMs inhibit PRRSV replication *in vitro* have been reviewed in detail (Bello-Onaghise et al., 2020), and include blocking PRRSV attachment and entry into cells, exerting activity against different stages of the PRRSV life cycle (i.e., interfering with viral RNA replication, viral particle assembly, and particle release), or acting as immunomodulators through cytokine regulation.

Since NADC30-like strains and HP-PRRSV-like strains appeared in swine farms, the commercially available PRRSV MLVs have provided limited protection or no protection against these genetically diverse isolates. Some bioactive compounds from TCMs remain popular in swine farms in China in different forms, including medicated feedstuff or oral or injected formulations.

#### Strict Biosecurity Measures

In China, a set of strict biosecurity measures were carried out in response to the African swine fever virus (ASFV), a highly contagious virus that is a major threat to domestic pigs and wild boars, which appeared in China in 2018. Since there was no effective vaccine or medicine that could be used to control the disease, biosecurity measure was the only way to prevent virus transmission. Risks, including people, pigs, vehicles and equipment, feed and water, as well as pests and air, were treated sterilely as soon as possible to keep this swine pathogen out of swine farms. The outcome was that the swine populations seemed to have remained healthy for a long time. Thus, many swine farmers have given their herds PRRSV vaccination. However, PRRSVs exist in most swine farms in China, and free use of PRRSV vaccination has led to HP-PRRSV-like or NADC30-like strains re-emerging in seemingly healthy populations since the end of 2020.

#### Inactivated Porcine Reproductive and Respiratory Syndrome Virus Vaccines

Different adjuvants (cytokines, chemical reagents, and bacterial products; detailed in Charerntantanakul, 2009), different administration routes (intramuscular or into the skin using dissolving microneedle patches), and different inactivation manners [binary ethyleneimine (BEI)-inactivated, beta-propiolactone (BPL)-inactivated, or ultraviolet (UV)-inactivated] have been tested to obtain inactivated PRRSV vaccine candidates to provide a better protective efficacy against clinical PRRSV isolate infection in piglets. Although some attempts could induce humoral immune responses, no successful cases have been reported that provided satisfactory protection efficacy to relieve clinical symptoms and reduce viremia and the pathological changes in the lung and other organs (Nilubol et al., 2004; Tabynov et al., 2016; Vreman et al., 2019, 2021).

There have been no reports about the efficacy of PRRSV-inactivated vaccines against NADC30-like strains in swine farms of China. However, following the appearance of NADC30-like strains in swine farms, sows have continued to be inoculated with the only commercial PRRSV-inactivated vaccine (CH-1a strain) in China. Currently, although the protective mechanism of the inactivated vaccine is unclear, it provides good protection for the gilts in PRRSV-affected swine farms.

## Implications and Conclusion

As a “mystery swine disease” that first appeared in swine farms (Pol et al., 1991; Stevenson et al., 1993; Wensvoort, 1993; Wills et al., 1997), PRRS has existed for more than 25 years. During this period, researchers have been working to determine the pathogenesis, immune mechanisms, and efficient control measures of PRRS, and have achieved milestones in clarifying the mechanism of immune responses, immunosuppression, and antiviral innate immunity evasion, as well as the development of MLV vaccines to relieve clinical symptoms, and reduce viremia and the pathological changes in the lung and other organs (for detailed information, see Lunney et al., 2016; Du et al., 2017; Wang et al., 2019, 2020). However, because of the recombination and variation characteristics, PRRSVs are still active in swine farms and have evolved into many variant strains, which have inflicted major losses on swine productivity.

In mainland China, since PRRS was first reported in 1996 (Guo et al., 1996), the virus has experienced two main variations. HP-PRRSV was the product of the first variation in May 2006, which represented a great challenge for the development of the pig industry in the following ∼10 years (Li et al., 2007; Tian et al., 2007; Tong et al., 2007). The second variation began in 2014 because of the invasion of the PRRSV NADC30 strain and recombination with the field PRRSV strains that were prevalent in the Chinese swine farms, causing various clinical outcomes from inapparent symptoms to severe symptoms, and representing new challenges for farms (Zhao et al., 2015; Zhou et al., 2015; Sun et al., 2016; Wang et al., 2018; Zhang et al., 2019). Although PRRS was placed under limited control in most swine farms, depending on the commercial MLV vaccines available before 2014, the invasion of PRRSV NADC30 and NADC30-like strain from 2014 onward broke the vulnerable PRRSV control balance. Routine and reliable control of PRRSV will require long-term exploration, and the recombination characteristics of PRRSV contribute to the emergence of variant virus strains, meaning their effects on swine farms must be monitored in the future.

## Author Contributions

HS and YY designed the structure and concept of the review. All authors wrote the article, contributed to the article, and approved the final version of the article.

## Conflict of Interest

The authors declare that the research was conducted in the absence of any commercial or financial relationships that could be construed as a potential conflict of interest.

## Publisher’s Note

All claims expressed in this article are solely those of the authors and do not necessarily represent those of their affiliated organizations, or those of the publisher, the editors and the reviewers. Any product that may be evaluated in this article, or claim that may be made by its manufacturer, is not guaranteed or endorsed by the publisher.

## Footnotes

1
https://www.ema.europa.eu/en/documents/product-information/aivlosin-epar-product-information_en.pdf
